# Competency of malaria laboratory diagnosis at national and provincial levels at the beginning of malaria post-elimination phase, China

**DOI:** 10.1186/s12936-024-04883-5

**Published:** 2024-02-26

**Authors:** He Yan, Mei Li, Zhi-gui Xia, Jian-hai Yin

**Affiliations:** National Institute of Parasitic Diseases, Chinese Center for Disease Control and Prevention (Chinese Center for Tropical Diseases Research), NHC Key Laboratory of Parasite and Vector Biology, WHO Collaborating Centre for Tropical Diseases, National Center for International Research on Tropical Diseases, Shanghai, 200025 China

**Keywords:** Malaria, Competency assessment, Microscopy, Nucleic acid amplification tests, Post-elimination

## Abstract

**Background:**

Qualified malaria diagnosis competency has contributed to the great achievement of malaria elimination in China. After eliminating malaria, it is still critical to the prevention of re-establishment of malaria transmission in China. This study was aimed to assess the malaria detection competency at national and provincial levels in China at the beginning of malaria post-elimination phase.

**Methods:**

In the present study, different competency assessment activities on the laboratory malaria diagnosis were carried out for national and provincial malaria diagnostic laboratories based on the WHO scoring schedules, including malaria microscopy or nucleic acid amplification tests (NAAT), at the beginning of malaria post-elimination phase (2021–2022) in China.

**Results:**

A total of 60 slides for malaria microscopy and 10 specimen for NAAT were included into the WHO External Quality Assessments of malaria parasite qualitative detection and species identification, and the scoring rate was 96.6% (microscopy: 171/177) and 85.0% (NAAT: 17/20), respectively. Moreover, 124 samples were included into the national NAAT quality assessment, and an accuracy of 87.9% (109/124) was found without significance among reference laboratories and non-reference laboratories.

**Conclusions:**

The findings suggest that there is still a need for sustained strengthening of malaria detection competency, particularly in the areas of parasite counting and detection of low-density parasitemia, to ensure prompt detection of the sources of infection and accurate identification of *Plasmodium* species, and contribute to case management and focus disposal, thereby effectively preventing the malaria re-establishment.

## Background

China had been certificated malaria-free by the World Health Organization (WHO) in 2021 [1, 2]. However, the global malaria burden remains heavy which caused around 400,000 deaths annually [3, 4]. Moreover, China still faces numerous challenges, including the large number of imported malaria cases per year, the continuous threat of border malaria, undetermined levels of asymptomatic infections and *Plasmodium falciparum* histidine-rich protein 2/3 gene deletions [5]. More importantly, introduced malaria cases caused by imported cases could occur in the presence of transmission conditions with malaria vector mosquitoes [6–8], if malaria diagnosis competency cannot identify all the sources of infection timely, resulting in the potential re-establishment of malaria transmission.

To date, five *Plasmodium* species including *Plasmodium falciparum*, *Plasmodium vivax*, *Plasmodium malariae*, *Plasmodium ovale* and *Plasmodium knowlesi*, are identified as human infectious pathogens, and also challenged by the potential zoonotic transmission of other malaria parasites [9]. Meanwhile, malaria microscopy, antigen-based rapid diagnostic test (RDT) and nucleic acid amplification tests (NAAT) are the most commonly used malaria diagnostic tools [10]. Among them, RDT cannot effectively distinguish all *Plasmodium* species except for *P. falciparum* and *P. vivax*. Furthermore, microscopy remains the gold standard for laboratory confirmation of malaria, although this method is highly experience-dependent and relatively less sensitive. In addition, nucleic acid detection technologies such as the polymerase chain reaction (PCR) with higher sensitivity and specificity have become an ideal means to identify *Plasmodium* species, and it has been considered as one of standard methods of Diagnosis of Malaria (WS259-2015) [11]. It is also required that each reported malaria case be confirmed by nucleic acid testing, and PCR is a necessary prerequisite for the establishment of a reference laboratory for malaria diagnosis in the China Malaria Diagnosis Reference Laboratory Network [12]. Although a qualified malaria microscopy competency was found in the subnational verification of malaria elimination in China, some deficiencies such as the identification of slides with low parasite density and more accurate species identification of *Plasmodium* spp., still needs to be improved [13]. Moreover, there is also a lack of external competency assessment of the nucleic acid detection capacity of malaria parasites in the China Malaria Diagnosis Reference Laboratory Network. And six provinces in the Chinese mainland still do not have provincial reference laboratories for malaria diagnosis to date [14].

Therefore, it is essential to continuously assess the malaria detection competency to ensure accurate identification of the sources of infection [15], which is one of the prerequisites of the 1-3-7 approach (in brief, the diagnosis, investigation and follow-up of malaria cases that must occur within 1, 3 and 7 days) in China [16], thereby effectively preventing the re-establishment of malaria transmission.

## Methods

### Samples preparation

A total of 60 blood slides were used in the present study, and they were from the WHO External Quality Assessment Programme worked with Research Institute for Tropical Medicine, WHO Collaborating Center for Malaria Diagnosis in the Philippines. Moreover, ten lyophilized blood samples were distributed by the WHO Global Malaria Programme with the UK National External Quality Assessment Scheme (UK NEQAS) Parasitology. In addition, 124 dried blood spots were prepared from residual blood samples referred from hospitals across the country, which were confirmed by the Chinese National Malaria Diagnosis Reference Laboratory.

### Competency assessment activities

During 2021 and 2022, the China National Malaria Diagnosis Reference Laboratory participated into four rounds of the WHO External Quality Assessment (EQA) Program for malaria microscopy. Moreover, one round of the WHO Malaria NAAT EQA was performed against the China National Malaria Diagnosis Reference Laboratory in 2021. In addition, one round of the NAAT quality assessment against malaria diagnosis laboratories in the provincial center for disease control and prevention (CDCs)/institute of parasitic diseases (IPDs) was held by the China National Malaria Diagnosis Reference Laboratory in 2021, and a total of 31 provincial laboratories participated into this assessment.

### Test samples and scoring

A total of 15 challenge slides per round were given to measure microscopists’ competency to detect and identify parasite species, and quantify parasites in the *P. falciparum*-positive slides. In the WHO malaria NAAT EQA, ten specimens containing five major human-infecting *Plasmodium* species at different parasite densities with or without negative samples in the formats of lyophilized blood and dried blood spot were distributed [10]. Furthermore, four dried blood spots with or without malaria parasites were distributed individually to 31 malaria diagnosis laboratories in the provincial CDCs/IPDs each. The scoring schedule for malaria microscopy and NAAT quality assessment from the WHO is provided in Table 1 [17, 18].

**Table 1:** Scoring schedule for malaria microscopy and NAAT quality assessment

Malaria microscopy^a^			NAAT		
Score	Species identification	Parasite counting	Score	Positive specimens	Negative specimens
3	Expected *Plasmodium* species	± 25% from reference count	2	Genus and species correctly identified	No *Plasmodium* nucleic acid detected
1	Expected *Plasmodium* species + other species	± 50% from reference count	1	Only *Plasmodium* genus identified	–
0	Any other response	< ± 25% or > ± 50% from reference count	0	(a) Correct genus but wrong species (b) Indeterminate result	Indeterminate result
Not scored	*P. knowlesi*	–	− 1	(a) No *Plasmodium* nucleic acid detected (b) *P. falciparum* nucleic acid not detected (relevant for labs doing *P. falciparum* identification only)	*Plasmodium* nucleic acid present

^a^The scoring schedule for malaria microscopy is from WHO External Quality Assessment Programme, with minor modification from Malaria Microscopy Quality Assurance Manual–Version 2 [17]

### Statistical analysis

The performance in different competency assessments was described as scores and accuracy through the descriptive statistics using Microsoft Excel 2010. The comparative analysis of performance among different groups was conducted with Pearson Chi-square tests or Fisher’s Exact Test using IBM SPSS Statistics (version 26). The level of significance was set at *P* < 0.05 (two-sided).

## Results

### Malaria microscopy EQA

A total of 60 slides including 24 *P. falciparum*-positive, 19 *P. vivax*-positive, 2 *P. malariae*-positive, 1 *P. knowlesi*-positive, 1 mixed positive of *P. falciparum* and *P. malariae*, and 13 *Plasmodium*-negative slides were assessed (Table 2). A total of 171 points (96.6%, 171/177) for species identification were received, but one *P. vivax*-positive slide (63 p/µL) was misdiagnosed as negative in the Round 1 of 2021, and one *P. vivax*-positive slide (13,920 p/µL) was misdiagnosed as *P. knowlesi*-positive in 2022.

**Table 2:** Parasite identification reported by the China National Malaria Diagnosis Reference Laboratory in Malaria Microscopy EQA, 2021–2022

Slide	Round 1 (2021)		Round 2 (2021)		Round 1 (2022)		Round 2 (2022)	
	Expected response	Score	Expected response	Score	Expected response	Score	Expected response	Score
1	Negative	3	Negative	3	Negative	3	Negative	3
2	Negative	3	Negative	3	Negative	3	Negative	3
3	Negative	3	Negative	3	Negative	3	Negative	3
4	*P. falciparum*	3	Negative	3	*P. falciparum*	3	*P. falciparum*	3
5	*P. falciparum*	3	*P. falciparum*	3	*P. falciparum*	3	*P. falciparum*	3
6	*P. falciparum*	3	*P. falciparum*	3	*P. falciparum*	3	*P. falciparum*	3
7	*P. falciparum*	3	*P. falciparum*	3	*P. falciparum*	3	*P. falciparum*	3
8	*P. falciparum*	3	*P. falciparum*	3	*P. falciparum*	3	*P. falciparum*	3
9	*P. falciparum*	3	*P. falciparum*	3	*P. falciparum*	3	*P. falciparum*	3
10	*P. vivax*	0	*P. falciparum*	3	*P. vivax*	3	*P. vivax*	3
11	*P. vivax*	3	*P. vivax*	3	*P. vivax*	3	*P. vivax*	3
12	*P. vivax*	3	*P. vivax*	3	*P. vivax*	3	*P. vivax*	3
13	*P. vivax*	3	*P. vivax*	3	*P. vivax*	3	*P. vivax*	3
14	*P. vivax*	3	*P. vivax*	3	*P. vivax*	3	*P. vivax*	0
15	*P. malariae*	3	*P. knowlesi*^a^	-	*P. falciparum *and* P. malariae*	3	*P. malariae*	3
Total		42		42		45		42

^a^Not scored, for educational purposes only

Moreover, 100% of six slides were quantified completely correct in Round 2 of 2021, while only 12 points (66.67%, 12/18) were received in Round 1 of 2021, 14 points (77.78%, 14/18) and 13 points (72.22%, 13/18) were received in Round 1 and Round 2 of 2022, respectively (*P* = 0.044, Fisher's Exact Test) (Table 3). Among them, all three slides in 2021 with relatively low parasite density (< 500 p/µL) were quantified correctly, but one slide at 396 p/µL in Round 2 in 2022 were failed, and one slide at 5783 p/µL and 20,133 p/µL each in Round 1 of 2021 were responded wrong, and one point was received individually from one slide at 485 p/µL and 3044 p/µL in Round 1 of 2022 and 10,956 p/µL in Round 2 of 2022 each (Table 3).

**Table 3:** Quantification of *Plasmodium falciparum* by the China National Malaria Diagnosis Reference Laboratory in Malaria Microscopy EQA, 2021–2022

Slide	Round 1 (2021)		Round 2 (2021)		Round 1 (2022)		Round 2 (2022)	
	Reference count (parasites/µL)	Score	Reference count (parasites/µL)	Score	Reference count (parasites/µL)	Score	Reference count (parasites/µL)	Score
1	333	3	400	3	439	3	396	0
2	661	3	101	3	485	1	775	3
3	4719	3	811	3	3044	1	1363	3
4	5783	0	875	3	5213	3	4663	3
5	16,509	3	4416	3	1295	3	6262	3
6	20,133	0	8424	3	5213	3	10,956	1
Total		12		18		14		13

### WHO NAAT EQA

In the WHO NAAT EQA, four *P. vivax*-positive, three *P. malariae*-positive, one *P. knowlesi*-positive and two *Plasmodium*-negative lyophilized specimens were assessed, and the QIAamp DNA Mini Kit (Qiagen, USA) followed by the nested PCR based on the *Plasmodium* 18S rRNA gene was applied. A total of 17 points (85.0%, 17/20) were received, including nine specimens (90.0%, 9/10) were detected and identified correctly, while no *Plasmodium* nucleic acid was detected in a *P. malariae*-positive specimen (2 × 10^4^ p/mL).

### National NAAT EQA

In the NAAT quality assessment held by the China National Malaria Diagnosis Reference Laboratory, and commercial DNA extraction kits (96.7%, 30/31) were used in most of the laboratories, and the nested PCR and real-time PCR were applied in 14 and 17 laboratories, respectively. As a result, there were 8, 3, and 1 laboratories responded one, two and three samples wrong respectively. And no significant differences (*P* = 0.282, χ^2^ = 1.514) were found in the scoring between reference laboratories and non-reference laboratories at provincial level.

In terms of species identification, a total of 124 samples (29 *P. falciparum*, 17 *P. ovale*, 16 *P. malariae*, 62 *Plasmodium* negative) were distributed, 87.9% (109/124) of samples were detected correctly, including 72.4% (21/29) of *P. falciparum*-positive samples, 100% (17/17) of *P. ovale*-positive samples, 93.8% (15/16) of *P. malariae*-positive samples and 90.3% (56/62) of negative samples, and there were significant differences among samples with different *Plasmodium* species or negative (*P* = 0.028, Fisher’s Exact Test).

No *Plasmodium* nucleic acid was detected in six *P. falciparum*-positive samples in six laboratories individually, and four samples were at low density (a total of seven *P. falciparum*-positive samples with low density). Moreover, another one *P. falciparum*-positive sample (low density) was misdiagnosed as *P. ovale* in one laboratory, and six *Plasmodium*-negative samples in five laboratories were misdiagnosed as *P. falciparum* (3), *P. vivax* (2) and *P. ovale* (1), respectively. Additionally, one sample was positive for *P. falciparum* or positive for *P. malariae* each was reported as mix infection of *P. falciparum* and *P. malariae*. Generally, the accuracy was much higher in detecting normal samples (81.0%, 17/21) than those with low density (37.5%, 3/8) in *P. falciparum*-positive samples, although no statistical significance (*P* = 0.067, Fisher’s Exact Test) was found.

## Discussion

Prompt and accurate diagnosis is an essential component of malaria control and elimination strategies, and it is even more important in areas after elimination but still at risk of retransmission where fever is less likely to be caused by malaria [5, 19]. Thus, only the source of infection (individuals infected with the malaria parasite or people with malaria) is timely and accurately detected and effectively managed, the retransmission of malaria can be prevented in such areas. Meanwhile, the quality control of the parasitological tests for malaria is critical to ensure the accuracy and comparability of malaria diagnosis [15]. In the present study, the competency assessments of malaria microscopy and NAAT at national or provincial levels were carried out and reported in a timely manner at the beginning of malaria post-elimination phase (2021–2022) in China. The competency of malaria parasite qualitative detection (positive or negative) and species identification by malaria microscopy and NAAT was qualified, while parasite counting by malaria microscopy and NAAT in detecting low-density samples were challenging in the China National Malaria Diagnosis Reference Laboratory and provincial laboratories respectively.

Recalling China’s efforts in laboratory diagnosis for malaria in the elimination phase, and the competency in a series of quality control activities, some challenges remain in maintaining and improving malaria laboratory testing capacity [14]. Fortunately, a high-level team of malaria microscopists with the WHO certificate has been developed, and malaria parasite qualitative detection and species identification by malaria microscopy has been quite good at the provincial level [13, 20], which is similar to the findings of this study. Meanwhile, there are still two major challenges of malaria microscopy, one of which is the species identification of other *Plasmodium* species rather than *P. falciparum*, especially the misidentification between *P. vivax* and *P. ovale* [21], and the other is the unstable performance of malaria parasite counting [20, 22]. In order to address these challenges, in addition to continuing to strengthen the competency training of microscopists, some automated systems or artificial intelligence tools are also considered to be introduced into the diagnosis of malaria [23–27]. Worryingly, there were still gaps in the competency of malaria microscopy in medical institutions and CDCs/IPDs below the provincial level in China [28–30]. All of the above is not conducive to the timely detection of the source of infection and poses a great challenge for prevention of reestablishment of malaria transmission in the country.

In terms of malaria parasite NAAT, PCR-based methods have been routinely used in the sample review of malaria parasites in national and provincial laboratories, but have not been fully extended to laboratories below the provincial level [12]. However, no malaria NAAT commercial kits (PCR kits) are available for clinical use in China currently, because all of them have not been approved by the National Medical Products Administration to date. Fortunately, a NAAT platform covering county-level medical and health institutions has been established and used to effectively respond to the coronavirus disease 2019 pandemic [31], and NAAT has been used as one of the diagnostic criteria for malaria [11]. Moreover, standards specific to malaria parasite nucleic acid detection using different methods are also continuously developed and implemented [32–34]. Therefore, quality assessment activities must be implemented to assess the reliablity of data and diagnosis when various NAAT methodologies and protocols used. These activities can be carried out following the global NAAT EQA scheme lauched by the WHO Global Malaria Programme worked with the UK NEQAS Parasitology and with technical experts [10, 18].

## Limitations

There are still some shortcomings in the present study, which needs to be further improved in the external competency assessment of malaria laboratory diagnosis in the future. First, the sample size is relatively small and insufficient to fully reflect the actual competency. Second, no *P. vivax* and *P. knowlesi* was included into the National NAAT EQA, which is not sufficient to fully reflect the capacity to identify *Plasmodium* species.

## Conclusions

Overall, a qualified competency of malaria parasite detection was found in the provincial and national malaria diagnosis laboratories through different competency assessment activities at the beginning of malaria post-elimination phase in China, but it is particularly challenged by parasite counting and detection of low-density parasitemia, indicating that sustained improvements of malaria laboratory diagnosis should be strengthened after elimination. The following aspects can be prioritized on the basis of the existing laboratory network. First, the awareness of quality control and quality assurance for malaria laboratory diagnosis should be further strengthened at all levels, and carried out different forms of quality assessments; second, targeted training should be carried out timely to improve the laboratory testing capacity, especially for the deficiencies found in the quality assessments; third, research and development of more appropriate techniques for parasite detection should be strengthened after elimination. All of these is to prevent the re-establishment of malaria transmission in China.

## Data Availability

All the data used to support the results of this research are available from Jian-hai Yin upon request.

## Abbreviations

NAAT: Nucleic acid amplification tests
WHO: World Health Organization
RDT: Rapid diagnostic test
PCR: Polymerase chain reaction
UK NEQAS: UK national external quality assessment scheme
EQA: External quality assessment
CDC: Center for disease control and prevention
IPD: Institute of parasitic diseases
